# The relationship between cognitive and affective control and adolescent mental health

**DOI:** 10.1002/jcv2.12204

**Published:** 2023-11-03

**Authors:** Savannah Minihan, Levi Kumle, Kate Maston, Debopriyo Bal, Aliza Werner‐Seidler, Helen Christensen, Susanne Schweizer

**Affiliations:** ^1^ School of Psychology University of New South Wales Sydney New South Wales Australia; ^2^ Department of Experimental Psychology University of Oxford Oxford UK; ^3^ Black Dog Institute University of New South Wales Sydney New South Wales Australia; ^4^ Discipline of Psychiatry and Mental Health University of New South Wales Sydney New South Wales Australia; ^5^ Department of Psychology University of Cambridge Cambridge UK

**Keywords:** adolescence, affective control, cognitive control, depression, mental health

## Abstract

**Background:**

Cognitive control problems have been implicated in the etiology and maintenance of mental health problems, including depression, in adults. Studies in adolescents have been more equivocal, with some showing changes in cognitive control in adolescents with mental health problems, whereas others fail to show an association. This study examines whether adolescent mental health is associated with *affective* control, the application of cognitive control in affective contexts, which shows more protracted development than cognitive control.

**Methods:**

The present study investigated the association of cognitive and affective control with depressive symptomatology and self‐reported diagnostic history of mental health problems in adolescents. The study included 1929 participants (*M*_age_ = 13.89) from the Future Proofing Study (*N* = 6,388, 11–16 years), who completed affective (incl., affective stimuli) and/or cognitive (incl., neutral stimuli) versions of a working memory (backward digit‐span) and/or shifting (card‐sorting) task at least once within 3 weeks of assessing mental health.

**Results:**

Poorer working memory was associated with greater depressive symptomatology in adolescents (*β* = −0.06, *p* = .004), similarly across cognitive and affective control conditions (*β* = −0.02, *p* = .269). Adolescents with self‐reported diagnostic history of mental health problems had significantly poorer shifting ability in affective compared to cognitive control conditions (*b* = 0.05, *p* = .010), whereas for adolescents with no self‐reported diagnoses, shifting ability did not differ between conditions (*b* = −0.00, *p* = .649).

**Conclusions:**

The present analyses suggest that working memory difficulties, in particular, may be associated with the experience of current depressed mood in adolescents. Problems with affective shifting may be implicated in a range of mental health problems in adolescents. Given the ubiquitous need for efficient cognitive functioning in daily life, enhancing cognitive and affective control in adolescents may be a promising means of improving functioning across a range of domains, including affective functioning, and by extension, adolescent mental health.

## Graphical abstract

Cognitive control problems have been implicated in the etiology and maintenance of mental health problems, including depression, in adults, whereas findings in adolescents have been more equivocal. Mental health problems in adolescents may be particularly associated with alterations in affective control. Our findings suggest that depressive symptoms in adolescents may be associated with working memory difficulties, whereas problems with affective shifting, which were associated with a history of self‐reported mental health diagnoses, may be implicated in a range of adolescent mental health problems.

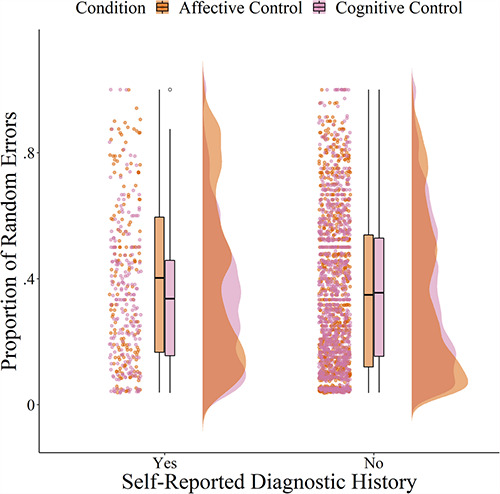

**Key points**

- Cognitive control problems have been implicated in the etiology and maintenance of mental health problems, including depression, in adults, whereas findings in adolescents have been more equivocal.
- Mental health problems in adolescents may be particularly associated with alterations in *affective* control.
- Depressive symptoms in adolescents were associated with working memory difficulties.
- Problems with affective shifting were associated with a history of self‐reported mental health diagnoses and may be implicated in a range of adolescent mental health problems.
- Enhancing cognitive and affective control in adolescents with mental health problems may be a promising means of improving daily functioning and potentially reducing mental health symptomatology.

## INTRODUCTION

Adolescence (10–24 years; Sawyer et al., 2018) is a period of vulnerability for the emergence of mental health problems; globally, about two thirds of all mental health disorders emerge before the age of 25 (Solmi et al., 2022). The incidence of depression, in particular, increases during adolescence (Thapar et al., 2012), with rates of depression in young people exacerbated in the context of the novel Coronavirus (COVID‐19) pandemic (Ludwig‐Walz et al., 2022). The majority of young people with mental health problems, however, do not seek mental health care support, for reasons including accessibility, affordability, and stigma (Gulliver et al., 2010; Radez et al., 2021). Of those young people who do receive support, up to 50% either don't respond to treatment or will experience a subsequent relapse of symptoms (Bear et al., 2020). Advancing our understanding of transdiagnostic and malleable features associated with mood and other common mental health problems in adolescence is therefore critical in order to identify novel avenues for prevention of, and early intervention for, adolescent mental health problems (Hagan et al., 2015). Here we examine the association between adolescent mental health, namely, depression and self‐reported diagnostic history of mental health problems, with cognitive and affective control.

Cognitive control can be defined as the capacity to engage in thoughts, emotions, and behaviors in line with our goals and to respond flexibly to changes in our environment (Snyder, 2013). Cognitive control develops throughout adolescence (Ferguson et al., 2021) and is driven by the experience‐dependent changes that occur during this critical phase in underlying brain regions, in particular, the prefrontal cortex (Luna et al., 2010). The experience‐dependent development of these brain regions suggests that cognitive control may be a potential malleable target for mental health interventions. Indeed, cognitive control problems have been implicated in the etiology and maintenance of common mental health problems, including depression, in adults (Dotson et al., 2020; McTeague et al., 2016; Snyder et al., 2015). Meta‐analytic evidence has shown that cognitive control problems are prevalent across a range of psychiatric presentations in adults, including depression, schizophrenia, bipolar disorder, obsessive compulsive disorder, post‐traumatic stress disorder, anxiety disorders, attention deficit hyperactivity disorder, and substance use disorders (Snyder et al., 2015). At the neural level, meta‐analytic evidence has shown common neural circuit disruptions in cognitive control networks in individuals with various mental health problems (McTeague et al., 2017). The pervasiveness of cognitive control problems across disorders has led researchers to suggest that impaired cognitive control may be a transdiagnostic risk factor for psychopathology (McTeague et al., 2016; Snyder et al., 2015; Zelazo, 2020).

At the mechanistic level, reduced cognitive control is theorized to bias thinking patterns and impair emotion regulation (LeMoult & Gotlib, 2019; Shi et al., 2019). Specifically, interpretation biases are associated with impaired inhibition of automatic negative thoughts in response to ambiguous information (Everaert et al., 2017). Poor emotion regulation in individuals with cognitive control problems are argued to be due to limited updating of regulatory strategies preventing individuals from switching to more situationally appropriate strategies (Joormann & Tanovic, 2015), as well as difficulties discarding mood‐congruent material from working memory (Koster et al., 2011). This shows that the different facets of executive functions, inhibition, shifting and working memory (Miyake & Friedman, 2012), that depend on cognitive control, may be differentially related to mental health problems.

However, the evidence for an association between cognitive control problems and adolescent mental health problems, is inconsistent. Some studies have shown cross‐sectional and prospective associations between cognitive control problems and general vulnerability to psychopathology, as well as with internalizing and externalizing symptomatology specifically, in adolescents (e.g., Romer & Pizzagalli, 2021; Snyder et al., 2019; Vedechkina et al., 2023; White et al., 2017). A recent systematic review similarly concluded that cognitive control problems among young people were associated with both general psychopathology and internalizing and externalizing disorders (Lynch et al., 2021). When considering depression specifically, some studies have similarly found evidence of poorer inhibition (e.g., Günther et al., 2011; Kyte et al., 2005), set‐shifting (e.g., Baune et al., 2012; Günther et al., 2011; Holler et al., 2014; Micco et al., 2009; Wilkinson & Goodyer, 2006), and updating (e.g., Baune et al., 2012; Holler et al., 2014) in depressed compared to healthy adolescents, whereas others have not (e.g., Favre et al., 2008; Matthews et al., 2008). Indeed, a recent meta‐analysis found no significant cross‐sectional differences between depressed and healthy adolescents (12–25 years) on measures of working memory, response inhibition, and set‐shifting (Goodall et al., 2018). However, when investigating the association between cognitive control and symptoms of depression prospectively, meta‐analytic evidence found that poorer cognitive control (incl. inhibition, shifting, and updating) during childhood or adolescence was significantly associated with more internalizing (incl., depression), as well as externalizing, symptoms later in life (Yang et al., 2022). Cognitive control problems in adolescents, then, may be more reliably associated with prospective rather than concurrent symptomatology. Indeed, whether cognitive control problems observed in mental health problems reflect underlying neurocognitive vulnerability (McTeague et al., 2016), phasic effects that fluctuate with symptoms, or scarring effects that worsen with symptom progression is debated in the literature (Allott et al., 2016; Hammar et al., 2022; Kriesche et al., 2022). Emerging evidence points toward a likely bi‐directional relationship between cognitive control problems and mental health problems in adolescence (Halse et al., 2022; Romer & Pizzagalli, 2021; but see, Brieant et al., 2022; Donati et al., 2021; Friedman et al., 2018).

Another potential account for the mixed findings on the association between cognitive control and adolescent mental health is that it is especially problems with cognitive control over affective material, *affective control*, that are related to poorer mental health. Affective control has been more reliably associated with depressive symptoms, as well as other mental health problems, in adolescents. Young people with depression or greater depressive symptomatology show reduced affective inhibition (e.g., Davidovich et al., 2016; Kilford et al., 2015), affective shifting (e.g., Lo & Allen, 2011; Wante et al., 2017), and affective working memory updating (e.g., Ladouceur et al., 2005; Tavitian et al., 2014) capacity compared to psychologically healthy adolescents. Beyond depression, affective control has been associated with several other mental health problems in adolescence, including dysphoria (e.g., Wante et al., 2018), anxiety (e.g., Ladouceur et al., 2009; Waters & Valvoi, 2009) and bipolar disorder (e.g., Passarotti et al., 2010). Together, these findings suggest that affective control, in particular, may be a transdiagnostic feature of mental health problems in adolescents.

The present study investigated the cross‐sectional associations of cognitive and affective control with adolescent mental health in the Future Proofing Study (FPS; Werner‐Seidler et al., 2020, 2023). The FPS is a longitudinal cohort study including 6388 adolescents and measures of cognitive and affective control, namely, a backward digit‐span (Schweizer, Leung, et al., 2019) and card‐sorting task (Schweizer, Parker, et al., 2020). Both tasks included a neutral and affective condition, providing an index of cognitive and affective control, respectively. Specifically, given the hypothesized transdiagnostic role of cognitive control problems in psychopathology, we investigated the association between cognitive and affective control and self‐reported diagnostic history of mental health problems. Given the mixed findings regarding the relationship between depressive symptomatology and cognitive control amongst adolescents, we additionally investigated the association between current depressive symptoms and cognitive and affective control. Examining the cross‐sectional associations between cognitive and affective control and adolescent depressive symptomatology allowed us to investigate the association of depressed mood states and cognitive functioning across neutral and affective task conditions. Examining associations with self‐reported diagnostic history, which includes individuals with an array of self‐reported psychiatric diagnoses, allowed us to investigate whether there was an association between cognitive and affective control and adolescent mental health across diagnoses.

Specifically, we investigated the following hypotheses. *Hypothesis 1:* Given research showing that affective control follows a more protracted course of development than cognitive control (Schweizer, Gotlib, & Blakemore, 2020), we predicted that performance on the backward digit‐span and card‐sorting tasks would be impaired in the affective relative to the cognitive control condition; that is, that adolescents would show poorer affective compared to cognitive control. We additionally predicted that heightened depressive symptomatology (*Hypothesis 2*) and self‐reported diagnostic history of mental health problems (*Hypothesis 3*) would be associated with impaired performance overall on the digit‐span and card‐sorting tasks, but more so in the affective control condition. In other words, we expected that adolescents with greater depressive symptomatology and/or who reported a history of diagnosed mental health problem(s) would show poorer cognitive and particularly affective control, relative to their peers with lower depressive symptoms/without self‐reported diagnostic history of mental health problems.

## METHOD

### Participants

Participants were drawn from the baseline assessment of the FPS (*N* = 6388), a longitudinal cohort study with an embedded cluster randomized controlled trial (cRCT; Werner‐Seidler et al., 2020, 2023). The FPS was conducted in 134 secondary schools located across Australia, with schools recruited via letter, email, and phone, as well as advertisements on social media and promotion at school conferences, between March 2019 and March 2022. In order to participate, schools were required to have a school counselor, psychologist, or wellbeing staff member onsite during the data collection phase. All adolescents enrolled in Year 8 (students aged 13–14 years) at participating schools were invited to take part in the study. Inclusion criteria included owning a smartphone with iOS or Android operating system with an active phone number. Baseline data was collected across three separate Year 8 cohorts: the 2019 cohort from August to September 2019; the 2020 cohort from August to November 2020; the 2021 cohort from April 2021 to March 2022. For further details on the design and cohort descriptives of the FPS, see Werner‐Seidler et al. (2023).

In order to be included in the present study, participants were required to have attempted the backward digit‐span task and/or card‐sorting task at least once within 3 weeks of completing the baseline questionnaire. The present study thus included 1929 participants (Table 1), with a mean age of 13.89 years (*SD* = 0.56), just over half of whom identified as female (55.05%). The number of young people born overseas was below the national average (16%; Australian Bureau of Statistics, 2022b) and the number of adolescents identifying as Aboriginal or Torres Strait Islander was just below the national average (5.6%; Australian Bureau of Statistics, 2022a). The sample of participants included in the present study did not differ to those participants in the wider FPS study who didn't complete any cognitive tasks on age, perceived socioeconomic status, or self‐reported diagnostic history of mental health problems; however, task completion was significantly associated with gender identity and depressive symptoms (see Appendix S1 and Table S1). Specifically, more females and fewer males than expected completed the tasks, and depressive symptoms were significantly higher in task‐completers compared to non‐completers.

**TABLE 1:** Summary of participant characteristics.

Participant characteristics	*n* (%)
**Gender identity**	
Female	1062 (55.05)
Male	751 (38.93)
Non‐binary	46 (2.39)
Other	30 (1.56)
Prefer not to say	40 (2.07)
**Country of birth**	
Australia	1773 (91.91)
Others	154 (7.98)
Missing	2 (0.10)
**Aboriginal and/or Torres Strait Islander**	
No	1811 (93.88)
Yes	85 (4.41)
Prefer not to say	33 (1.71)
**Perceived socioeconomic status**	
Low	151 (7.83)
Medium	632 (32.76)
High	902 (46.76)
Prefer not to say	244 (12.65)
**Self‐reported diagnostic history**	
Generalized anxiety disorder	171 (8.86)
Social anxiety disorder	116 (6.01)
Attention deficit hyperactivity disorder	97 (5.03)
Major depression	65 (3.37)
Obsessive compulsive disorder	29 (1.50)
Panic disorder	28 (1.45)
Post‐traumatic stress disorder	23 (1.19)
Separation anxiety disorder	22 (1.14)
Substance use disorder	2 (0.10)
Schizophrenia/Psychosis	2 (0.10)
Alcohol user disorder	0 (0)
No diagnosis	1605 (83.20)

### Measures

See Werner‐Seidler et al. (2023) for a full list of measures included in the FPS. Only those measures included in the present study are described below. For descriptives and bivariate correlations, see Table 2.

**TABLE 2:** Descriptives and bivariate correlations.

Variable	*M*	*SD*	1	2	3	4	5
1. Depressive symptoms	7.86	6.36					
2. Self−Reported diagnostic history			0.29**				
			[0.25, 0.33]				
3. Affective working memory	4.17	1.23	−0.09**	−0.03			
			[−0.14, −0.03]	[−0.09, 0.02]			
4. Cognitive working memory	4.25	1.23	−0.04	−0.01	0.29**		
			[−0.10, 0.02]	[−0.07, 0.04]	[0.21, 0.35]		
5. Affective shifting	0.35	0.25	0.01	0.08*	−0.11**	−0.15**	
			[−0.05, 0.08]	[0.01, 0.14]	[−0.18, −0.03]	[−0.22, −0.07]	
6. Cognitive shifting	0.36	0.23	0.00	−0.02	−0.13**	−0.24**	0.44**
			[−0.06, 0.06]	[−0.08, 0.04]	[−0.20, −0.06]	[−0.30, −0.17]	[0.37, 0.50]

*Note*: Table 2 shows descriptive statistics and bivariate correlations between depressive symptoms, self‐reported diagnostic history of mental health problems, affective and cognitive working memory, and affective and cognitive shifting. Depressive symptoms were assessed with the 9‐item PHQ‐A; higher scores indicate greater depressive symptomatology (Johnson et al., 2002). Self‐reported diagnostic history was assessed with a multi‐response item, where participants indicated whether they had ever been diagnosed by a professional with a mental health problem, selecting all the apply from the following: major depression, social anxiety disorder/social phobia, generalized anxiety disorder, obsessive compulsive disorder, panic disorder, separation anxiety disorder, alcohol use disorder, substance use disorder, attention deficit hyperactivity disorder, post‐traumatic stress disorder, schizophrenia/psychosis, none of the above. For the correlational analyses, this variable was dummy coded: 0 = participant reported having not been diagnosed with a mental health problem; 1 = participant reported having been diagnosed with a mental health problem. Working memory was operationalized as max span level achieved on a backward digit‐span task (Schweizer, Leung, et al., 2019). The task included a cognitive control condition, in which digits were superimposed over neutral images, and an affective control condition, in which digits were superimposed over negative images. Shifting ability was operationalized as proportion of random errors on a card‐sorting task, with higher scores indicating poorer shifting ability (Schweizer, Leung, et al., 2019). The task included a cognitive control and affective control condition, with task condition manipulated by inclusion of a sorting rule according to shape (cognitive control condition) or emotional expression (affective control condition). For correlations between continuous variables, Pearson's correlations are reported. For correlations with self‐reported diagnostic history, point biserial correlations are reported. Where participants completed the same task condition in session 1 and 2, their data was averaged across sessions.

#### Demographics

Participants provided a series of demographic measures, including age, gender identity, country of birth, Aboriginal and/or Torres Strait Islander status, perceived socioeconomic status, and self‐reported history of mental health diagnoses. Perceived socioeconomic status was assessed with a single item: participants were asked “how well off do you think your family is?”, with response options “Not at all”, “Not very”, “Fairly”, “Rather”, “Very”, and “Prefer not to say”. Low perceived socioeconomic status was operationalized as response options “Not at all” and “Not very”, medium perceived socioeconomic status was operationalized as response option “Fairly”, and high perceived socioeconomic status was operationalized as response options “Rather” and “Very”; “Prefer not to say” was coded as missing. Self‐reported diagnostic history of mental health disorders was assessed with a multi‐response item, where participants indicated whether they had ever been diagnosed by a professional with a mental health problem, selecting all that apply from the following: major depression, social anxiety disorder/social phobia, generalized anxiety disorder, obsessive compulsive disorder, panic disorder, separation anxiety disorder, alcohol use disorder, substance use disorder, attention deficit hyperactivity disorder, post‐traumatic stress disorder, schizophrenia/psychosis, none of the above.

#### Depressive symptoms

Current depressive symptomatology was assessed with the 9‐item Patient Health Questionnaire for Adolescents (PHQ‐A; Johnson et al., 2002). The PHQ‐A is an adaptation of the PHQ‐9 for adolescents, and requires participants to indicate the frequency they have experienced symptoms such as “Feeling down, depressed, irritable, or hopeless” over the previous 2 weeks on a scale ranging from 0 (*Not at all*) to 3 (*Nearly every day*). The PHQ‐A has demonstrated good psychometric properties (Johnson et al., 2002), and demonstrated good internal consistency in the current study (Revell's total omega = 0.91).

#### Cognitive and affective control

***Working Memory.*** Working memory was assessed with a backward digit‐span task (Schweizer, Leung, et al., 2019), in which participants were presented with digits (1500 ms) in serial order to be recalled in reverse order. The task included a cognitive control condition, in which digits were superimposed over neutral images, and an affective control condition, in which digits were superimposed over negative images. Images were taken from the Geneva Affective Picture Database (Dan‐Glauser & Scherer, 2011). Presentation order of the cognitive and affective control conditions was randomized. The task commences with two digits per trial, with each span level presented twice (i.e., two trials per span level). Participants are required to answer at least one trial per span level correctly in order to progress to the next span level. Performance on the task was operationalized as max span level achieved. Outlier values, defined as max span levels greater than 3 standard deviations above or below the sample mean span level (collapsed across cognitive and affective control conditions), were removed from analyses. This resulted in the exclusion of 18 datapoints from the cognitive control condition and 13 datapoints from the affective control condition. Both the affective and cognitive control task conditions showed acceptable (Clark et al., 2022; Hedge et al., 2018) reliability (average intraclass correlation (ICC) = 0.50 for the cognitive control condition and 0.52 for the affective control condition).

During the data cleaning process, an error was identified in how the data from the backward digit‐span task was processed and recorded on the Future Proofing app. Specifically, on trials in which the presented digit was a palindrome, the app would record the participant's response as an error, even if it was entered correctly. This meant that if the participant had answered the other trial in that span level incorrectly, the task would have terminated at that span level. We recoded accuracy for all relevant trials from incorrect to correct, however, we are unable to correct for the fact that for some participants the task terminated early, and thus their recorded span level may be inaccurate. This applied to 236 of 2564 datapoints included in analyses. However, removing these datapoints from analyses does not change the pattern of results. Therefore, we did not exclude these datapoints from results reported here.

***Shifting.*** Shifting ability was assessed using a card‐sorting task (Schweizer, Parker, et al., 2020), adapted from the Madrid Card Sorting Task (Barceló, 2003). In this task, participants are dealt a card, which they are required to assign to one of four decks according to three possible sorting rules: card color, number of items, and shape (cognitive control condition) or emotional expression (affective control condition). Presentation order of the cognitive and affective control conditions was randomized. The sorting rule switched randomly after 6–9 trials. If participants did not respond on a trial within 30 s, their response was recorded as an error. Performance on the task was operationalized as accuracy (i.e., proportion of random errors) and reaction time on color and number trials. Random errors occur when a participant responds incorrectly on any trial in the series after the initial two trials (which are needed to establish the correct sorting rule). Only performance on color and number trials was included, as the perceptual difficulty of the third sorting rule (i.e., shape or emotional expression) differed as a function of task condition. That is, emotional expression classification (affective control condition) is perceptually more complex and demanding than classifying shapes (cognitive control condition; Schweizer, Parker, et al., 2020). Reaction times and accuracy greater than 3 standard deviations above or below the sample mean reaction time/accuracy (collapsed across cognitive and affective control conditions) were removed from analyses. For the reaction time data, this resulted in the exclusion of 16 datapoints for the cognitive control condition and 27 datapoints for the affective control condition. No datapoints were excluded from the accuracy data. Both the affective and cognitive control conditions showed good reliability (average ICC = 0.73 for the cognitive control condition and 0.64 for the affective control condition).

### Procedure

Ethics approvals were obtained from the University of New South Wales Human Research Ethics Committee (HC180836), the State Education Research Applications Process for the New South Wales Department of Education (SERAP2019201), and relevant Catholic Schools Dioceses across Australia. Participating schools were provided with recruitment materials (available in English, Arabic, and Chinese) to share with Year 8 families, inviting parents/guardians to provide informed consent (online, by paper, or by phone) for their child's participation. Prior to taking part in the study, all participants required informed consent from a parent or guardian.

The baseline questionnaire was completed during a group session at school facilitated (57% in person) by the study team. Sessions that coincided with COVID‐19 lockdown restrictions were facilitated remotely over zoom, with participants joining either from school (41%) or home (2%). Participants were first introduced to the study, after which they logged in to the study website, where they provided informed consent and completed the baseline questionnaire individually and without input from the study team. Next, participants downloaded the Future Proofing app to complete, amongst other tasks (see Werner‐Seidler et al., 2023), the backward digit‐span and card‐sorting task, which concluded the baseline session.

Following the baseline assessment session, participants were encouraged to access the app and complete the cognitive tasks as many times as they wished in their own time (though for the present study, only data from tasks completed within 3 weeks of the baseline questionnaire were included). All participants received an AUD$20 gift card to reimburse personal data costs. For details on the cRCT aspect of the FPS, see Werner‐Seidler et al. (2023).

### Statistical analyses

First, data on the cognitive tasks were aggregated and grouped into sessions, separately for the backward digit‐span and card‐sorting tasks. A session could include data from the cognitive and/or affective control conditions of each task, with the majority of participants completing only one condition (i.e., cognitive or affective) of the task within a single session. For the present analyses, in order to reduce the influence of practice effects, only data from sessions one and two were included (for a distribution of task conditions completed across sessions see Appendix S2).

In order to investigate the relationship between cognitive and affective control and depressive symptoms, a series of linear mixed effects models were specified, all of which controlled for session number (coded numerically: session one = 1, session two = 2) and included a random effect for participant ID. All models were repeated twice; once with max span level on the backward digit‐span task as the outcome variable (i.e., working memory) and once with proportion of random errors on the card‐sorting task as the outcome variable (i.e., shifting ability).

In order to investigate potential differences between cognitive and affective control, condition (i.e., cognitive or affective control condition) was included as a fixed effect. Condition was contrast‐coded, such that cognitive control condition = −1, affective control condition = 1. Next, to determine whether cognitive and affective control were differentially associated with depressive symptomatology, an interaction term between condition and depressive symptoms was included. Depressive symptoms were mean‐centered. In a separate model, an interaction term between condition and self‐reported diagnostic history of mental health problems was included. Self‐reported diagnostic history was contrast‐coded, such that no self‐reported diagnostic history = −1, self‐reported diagnostic history = 1. Given the relatively low levels of self‐reported diagnoses reported in this sample (16.80%), we did not break this down into disorder type, as doing so would reduce our power to test effects between disorders. This approach also maintains consistency with how other studies into the association between psychiatric history and affective control have been conducted (e.g., Schweizer & Dalgleish, 2016). Sensitivity analyses controlling for age, self‐identified gender, and perceived socioeconomic status were conducted (see Tables S2 and S3).

A Bonferroni‐corrected significance threshold of *α* ≤ 0.0125 was adopted, to account for the inclusion of two mental health variables (i.e., depressive symptomatology and self‐reported diagnostic history of mental health problems) across two outcomes (working memory and shifting accuracy). All analyses were conducted in R Studio version 4.1.2. For specific packages used, see Appendix S3. The data analysis script is available on the Open Science Framework (https://osf.io/pndur/).

## RESULTS

Linear mixed effects models revealed that, contrary to Hypothesis 1, neither working memory, as measured by max span achieved on the backward digit‐span task (Table 3, Model 1), nor shifting ability, as measured by proportion of random errors on the card‐sorting task (Table 4, Model 1), was significantly impaired in the affective relative to the cognitive control condition. That is, when operationalized as working memory or shifting ability, adolescents' cognitive control did not significantly differ from their affective control.

**TABLE 3:** The relationship between cognitive and affective working memory, depressive symptoms, and self‐reported diagnostic history of mental health problems.

	Model 1: Working memory					Model 2: Working memory					Model 3: Working memory				
	*b*	*SE*	*95% CI*	*β*	*p*	*b*	*SE*	*95% CI*	*β*	*p*	*b*	*SE*	*95% CI*	*β*	*p*
Intercept	3.89	0.07	3.76 to 4.02	−0.01	<0.001	3.90	0.07	3.76 to 4.03	−0.01	<0.001	3.86	0.07	3.72 to 4.00	−0.03	<0.001
Condition	−0.05	0.02	−0.09 to −0.00	−0.04	0.038	−0.05	0.02	−0.09 to −0.00	−0.04	0.040	−0.06	0.03	−0.12 to −0.00	−0.05	0.042
Session number	**0.25**	**0.05**	**0.15** to **0.35**	**0.09**	**<0.001**	**0.25**	**0.05**	**0.15** to **0.34**	**0.09**	**<0.001**	**0.25**	**0.05**	**0.15** to **0.34**	**0.09**	**<0.001**
Depressive symptoms						**−0.01**	**0.00**	**−0.02** to **−0.00**	**−0.06**	**0.004**					
Condition × depressive symptoms						−0.00	0.00	−0.01 to 0.00	−0.02	0.269					
Self‐reported diagnostic history											−0.04	0.04	−0.12 to 0.03	−0.04	0.229
Condition × self‐reported diagnostic history											−0.02	0.03	−0.08 to 0.04	−0.02	0.448
Random effects															
σ^2^	1.10					1.10					1.10				
τ_00_	0.46 _ID_					0.45 _ID_					0.46 _ID_				
ICC	0.29					0.29					0.30				
N	1639 _ID_					1639 _ID_					1639 _ID_				
Observations	2564					2564					2564				
Marginal *R* ^2^/Conditional *R* ^2^	0.009/0.301					0.014/0.302					0.010/0.302				

*Note*: Table 3 shows the results of a series of linear mixed effects models investigating the relationship between cognitive and affective working memory, depressive symptoms, and self‐reported diagnostic history of mental health problems. Working memory was operationalized as max span level achieved on a backward digit‐span task (Schweizer, Leung, et al., 2019). The task included a cognitive control condition, in which digits were superimposed over neutral images, and an affective control condition, in which digits were superimposed over negative images. This variable was contrast‐coded for analyses: cognitive control condition = −1, affective control condition = 1. Depressive symptoms were assessed with the 9‐item PHQ‐A and were mean‐centered for analyses; higher scores indicate greater depressive symptomatology (Johnson et al., 2002). Self‐reported diagnostic history was assessed with a multi‐response item, where participants indicated whether they had ever been diagnosed by a professional with a mental health problem, selecting all the apply from the following: major depression, social anxiety disorder/social phobia, generalized anxiety disorder, obsessive compulsive disorder, panic disorder, separation anxiety disorder, alcohol use disorder, substance use disorder, attention deficit hyperactivity disorder, post‐traumatic stress disorder, schizophrenia/psychosis, none of the above. This variable was contrast‐coded for analyses: −1 = participant reported no mental health diagnoses; 1 = participant reported having been diagnosed with a mental health problem. Session number was modeled numerically (session one = 1, session two = 2). Bold values denote significant effects at *p* ≤ 0.0125.

**TABLE 4:** The relationship between cognitive and affective shifting, depressive symptoms, and self‐reported diagnostic history of mental health problems.

	Model 1: Shifting ability					Model 2: Shifting ability					Model 3: Shifting ability				
	*b*	*SE*	*95% CI*	*β*	*p*	*b*	*SE*	*95% CI*	*β*	*p*	*b*	*SE*	*95% CI*	*β*	*p*
Intercept	0.46	0.01	0.44 to 0.49	0.00	<0.001	0.46	0.01	0.44 to 0.49	0.00	<0.001	0.47	0.01	0.44 to 0.50	0.03	<0.001
Condition	0.00	0.00	−0.01 to 0.01	0.01	0.534	0.00	0.00	−0.01 to 0.01	0.01	0.539	0.01	0.01	0.00 to 0.02	0.05	0.032
Session number	**−0.08**	**0.01**	**−0.10** to **−0.07**	**−0.16**	**<0.001**	**−0.08**	**0.01**	**−0.10** to **−0.07**	**−0.16**	**<0.001**	**−0.08**	**0.01**	**−0.10** to **−0.07**	**−0.16**	**<0.001**
Depressive symptoms						0.00	0.00	−0.00 to 0.00	0.00	0.928					
Condition x depressive symptoms						0.00	0.00	−0.00 to 0.00	0.01	0.452					
Self‐reported diagnostic history											0.01	0.01	−0.01 to 0.02	0.04	0.267
Condition x self‐reported diagnostic history											**0.01**	**0.01**	**0.00** to **0.03**	**0.06**	**0.011**
Random effects															
σ^2^	0.03					0.03					0.03				
τ_00_	0.03 _ID_					0.03 _ID_					0.03 _ID_				
ICC	0.48					0.48					0.48				
N	1406 _ID_					1406 _ID_					1406 _ID_				
Observations	2316					2316					2316				
Marginal *R* ^2^/Conditional *R* ^2^	0.024/0.497					0.024/0.497					0.026/0.498				

*Note*: Table 4 shows the results of a series of linear mixed effects models investigating the relationship between cognitive and affective shifting, depressive symptoms, and self‐reported diagnostic history of mental health problems. Shifting ability was operationalized as proportion of random errors on a card‐sorting task, with higher scores indicating poorer shifting ability (Schweizer, Leung, et al., 2019). The task included a cognitive control and affective control condition, with task condition manipulated by inclusion of a sorting rule according to shape (cognitive control condition) or emotional expression (affective control condition). This variable was contrast‐coded for analyses: cognitive control condition = −1, affective control condition = 1. Depressive symptoms were assessed with the 9‐item PHQ‐A and were mean‐centered for analyses; higher scores indicate greater depressive symptomatology (Johnson et al., 2002). Self‐reported diagnostic history was assessed with a multi‐response item, where participants indicated whether they had ever been diagnosed by a professional with a mental health problem, selecting all the apply from the following: major depression, social anxiety disorder/social phobia, generalized anxiety disorder, obsessive compulsive disorder, panic disorder, separation anxiety disorder, alcohol use disorder, substance use disorder, attention deficit hyperactivity disorder, post‐traumatic stress disorder, schizophrenia/psychosis, none of the above. This variable was contrast‐coded for analyses: −1 = participant reported no mental health diagnoses; 1 = participant reported having been diagnosed with a mental health problem. Session number was modeled numerically (session one = 1, session two = 2). Bold values denote significant effects at *p* ≤ 0.0125.

### Association between depressive state and cognitive and affective control

There was a significant main effect of current depressive symptoms on working memory (Table 3, Model 2), such that higher depressive symptomatology was associated with lower working memory. However, this effect did not differ as a function of task condition (Table 3, Model 2); that is, individuals with greater depressive symptoms did not show significantly poorer affective relative to cognitive working memory. The effect of depressive symptoms survived sensitivity analysis for age (Table S2, Model 1), however, this effect did not reach our Bonferroni‐corrected significance level when controlling for gender identity (Table S2, Model 2) or perceived socioeconomic status (Table S2, Model 3). Conversely, depressive symptoms were not significantly associated with cognitive or affective shifting ability (Table 4, Model 2).

### Association between self‐reported diagnostic history of mental health problems and cognitive and affective control

**FIGURE 1:**
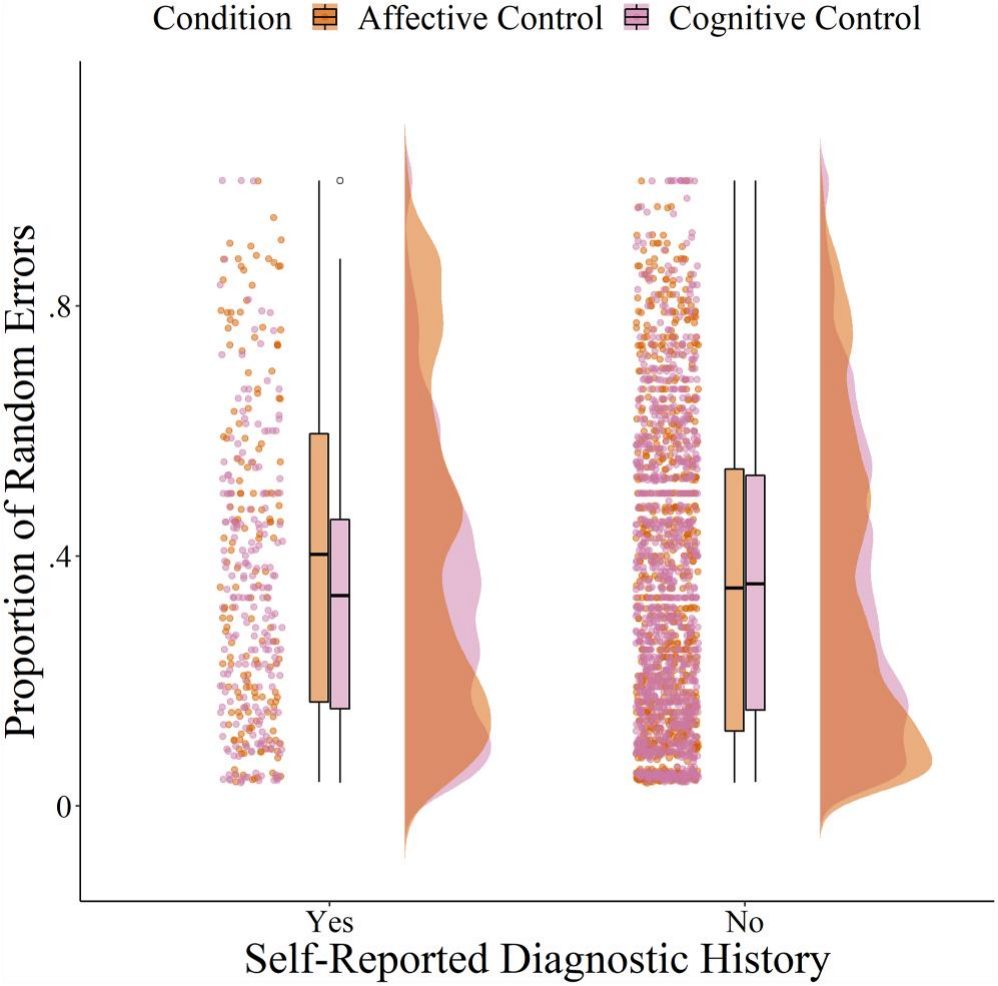
The relationship between cognitive and affective shifting and self‐reported diagnostic history of mental health problems. The raw data from the set‐shifting task plotted as a function of self‐reported diagnostic history of mental health problems and task condition. Task condition was manipulated by inclusion of a sorting rule according to shape (cognitive control condition) or emotional expression (affective control condition). Self‐reported diagnostic history was assessed with a multi‐response item, where participants indicated whether they had ever been diagnosed by a professional with a mental health problem, selecting all the apply from the following: major depression, social anxiety disorder/social phobia, generalized anxiety disorder, obsessive compulsive disorder, panic disorder, separation anxiety disorder, alcohol use disorder, substance use disorder, attention deficit hyperactivity disorder, post‐traumatic stress disorder, schizophrenia/psychosis, none of the above. Shifting ability was operationalized as proportion of random errors on a set‐shifting task, with higher scores indicating poorer shifting ability (Schweizer, Leung, et al., 2019).

Self‐reported diagnostic history of mental health problems was not significantly associated with cognitive or affective working memory (Table 3, Model 3). While there was no significant main effect of self‐reported diagnostic history of mental health problems on shifting ability, there was a significant interaction between self‐reported diagnostic history and task condition (Table 4, Model 3; Figure 1). Simple effects analyses revealed that, for individuals without a self‐reported diagnosis, shifting ability did not significantly differ between the cognitive and affective task conditions (*b* = −0.004, *SE* = 0.01, *95% CI* [−0.02 to –0.01], *p* = .649). In contrast, adolescents who reported having received a psychiatric diagnosis had significantly poorer shifting ability in the affective compared to the cognitive control condition (*b* = 0.05, *SE* = 0.02, *95% CI* [0.01–0.09], *p* = 0.010; Figure 1). The effect remained significant in sensitivity analysis including age (Table S3, Model 1), but did not reach our Bonferroni‐corrected significance when controlling for gender identity (Table S3, Model 2) or perceived socioeconomic status (Table S3, Model 3).

For the card‐sorting task, reaction time was available, which allowed us to examine whether the observed interaction term for shifting ability was a simple speed‐accuracy trade‐off. There was no evidence to support a speed‐accuracy trade‐off. That is, while overall participants were significantly faster in the cognitive control compared to the affective control condition (Table S4, Model 1), the effect of task condition did not differ across self‐reported diagnostic history status (Table S4, Model 2).

## DISCUSSION

The present study assessed the cross‐sectional relationship between cognitive and affective control with depressive symptoms and self‐reported diagnostic history of mental health problems in a large sample of adolescents. Contrary to our hypotheses, we found that, overall, working memory and shifting accuracy did not significantly differ across cognitive and affective control conditions, suggesting that, in this sample, affective working memory and shifting may have reached maturity by early‐ to mid‐adolescence (Schweizer, Gotlib, & Blakemore, 2020). Greater depressive symptomatology was associated with poorer working memory, similarly across cognitive and affective control conditions. Conversely, depressive symptoms were not associated with cognitive or affective shifting ability. Shifting ability differed as a function of task condition and self‐reported diagnostic history. That is, while individuals without a self‐reported psychiatric diagnosis did not show significantly different shifting ability across the cognitive and affective control conditions, individuals with a self‐reported history of psychiatric diagnosis had significantly poorer shifting ability in the affective compared to the cognitive control condition. This was not merely a speed‐accuracy trade‐off, as there was no significant interactive effect of condition and self‐reported diagnostic history on shifting reaction times. The present results thus suggest that facets of cognitive and affective control may be differentially associated with mental health problems in adolescence. While depressive states appear associated with working memory difficulties across neutral and affective contexts, affective shifting difficulties, which were associated with self‐reported diagnostic history of mental health problems, may be associated with a range of adolescent mental health problems.

Past research in adults has implicated alterations in cognitive control, including reduced working memory and shifting abilities, as a potential risk and maintaining factor for depressive symptoms (Snyder, 2013). While similar findings have been observed in adolescents (e.g., Wagner et al., 2015; but see Goodall et al., 2018), the results of the present study suggest that cognitive control deployed across different facets of executive functioning (Miyake & Friedman, 2012) may not be uniformly associated with depressive symptoms in adolescents. Our findings suggest that working memory, in particular, may be implicated in the experience of depressive symptoms in adolescents. Theoretical accounts of the role of impaired working memory in depression suggest that problems with working memory may contribute to difficulties discarding mood‐congruent material from working memory (Koster et al., 2011), in turn leading to perseverative styles of thinking, such as rumination (Joormann & Tanovic, 2015). While the present cross‐sectional findings cannot distinguish underlying neurocognitive vulnerability from phasic or scarring effects, they do highlight working memory as a potentially promising target for interventions for adolescent depression. Indeed, there has recently been a proliferation of research investigating the impact of working memory training on mental health symptoms, with promising results observed amongst adolescents, including improved depressive symptomatology following training (for a review, see Edwards et al., 2022).

Conversely, in the present sample, shifting ability was not associated with depressive symptomatology. It is possible that difficulties with shifting are associated with more severe depressive symptoms; in the present study, participants were, on average, experiencing mild depressive symptoms. In support of this speculation, compared to an outpatient control group, adolescent inpatients with both major and minor depression exhibited lower working memory updating, whereas only those with major depression exhibited poorer set‐shifting (Holler et al., 2014). In further support of this speculation, we found that self‐reported diagnostic history of mental health problems, which may capture those with more severe (current or remitted) symptomatology, was associated with shifting ability. Specifically, those participants who reported that they had previously been diagnosed with a mental health problem showed poorer affective compared to cognitive shifting ability; whereas in participants who did not report any psychiatric diagnoses, cognitive and affective shifting ability did not significantly differ. Applying affective control may therefore be particularly challenging for adolescents with a history of mental health problems. This finding provides support to conceptualizations of affective control as a transdiagnostic factor associated with mental health problems in adolescents (Schweizer, Parker, et al., 2020). Difficulties with affective shifting, in particular, have been proposed to contribute to problems shifting between prepotent maladaptive and situationally appropriate emotion regulation strategies (Schweizer, Gotlib, & Blakemore, 2020; Schweizer, Parker, et al., 2020). Emotion regulation itself is well‐established as a transdiagnostic risk factor for mental health problems (Aldao et al., 2010).

The interactive effect of task condition and self‐reported diagnostic history on shifting but not working memory may be accounted for by task‐related differences in the present study. The card‐sorting task included socially‐relevant stimuli (i.e., emotional facial expressions) that are frequently encountered in daily life, whereas the backward digit‐span task included mixed, often non‐social affective material (e.g., images of spiders and snakes). While speculative, it is possible that, for adolescents with mental health problems, the exertion of affective control may be more impaired in the context of more socially‐relevant affective distractors, such as emotional facial expressions. Indeed, adolescence as a development period is characterized by heightened sensitivity to social information (Blakemore & Mills, 2014). This speculation similarly may apply to the lack of interaction effect observed between depressive symptomatology and task condition on working memory; that is, the stimuli may not have been sufficiently personally salient to detract affective control resources from task goals amongst adolescents with heightened depressive symptomatology. Future research using social affective stimuli across different cognitive and affective control tasks is needed to test these speculations.

Despite the strengths of this study, including a large sample size and the inclusion of two cognitive and affective control tasks, a number of limitations should be considered when interpreting the present results. First, as aforementioned, the present study assessed the cross‐sectional relationship between cognitive and affective control with depressive symptoms and self‐reported diagnostic history of mental health problems amongst adolescents, therefore precluding any causal or directional conclusions. A second limitation was our measure of diagnostic history, which relied on self‐report data. It is unlikely that this variable accurately captured all adolescents who meet diagnostic criteria for a mental health problem, as many individuals who may meet criteria for a diagnosis may never seek or receive mental health support (Gulliver et al., 2010; Radez et al., 2021). This variable also relied on adolescents accurately remembering any diagnoses they had received, as well as adolescents being accurately diagnosed and accurately informed of any diagnoses received. This can introduce memory recall biases and inaccuracies, as well as being reliant on the accuracy of the information being provided, which, in mental health primary care settings, is not always the case (Davis et al., 2016; Larvin et al., 2019; Smith et al., 2021). Moreover, while the level of self‐reported diagnoses reported in this sample was generally representative of levels observed in the population and not unusually low for this age group, we did not break this down into disorder type and compare across diagnoses, which would have compromised the reliability of the data. Therefore, we were unable to determine whether there was a particular diagnosis driving the association between self‐reported diagnostic history and shifting ability. Large‐scale, longitudinal studies, assessing the application of cognitive and affective control as well as symptoms of different mental health disorders at several timepoints, are needed in order to determine the potential mechanistic and transdiagnostic role of cognitive and affective control in adolescent mental health problems. We also did not have sufficient information to model current versus remitted diagnostic status. Arguably, though, if the associations were largely being driven by the impact of current symptom levels on cognitive functioning, we would have observed more consistent performance decrements across both working memory and shifting in those with heightened depressive symptoms or self‐reported diagnostic history. In addition, because the majority of participants did not complete both the cognitive and affective control conditions of the tasks in the same session, we were unable to compute an *affective* control index (i.e., a proportional difference score indexing task performance in affective relative to cognitive conditions), which has typically been used in previous studies investigating the relationship between affective control and mental health amongst adolescents (e.g., Schweizer, Parker, et al., 2020). Therefore, differences in operationalization of affective control should be considered when comparing the present results to the literature in the field. Further, our sensitivity analyses suggested that gender identity and perceived socioeconomic status may play a role in the observed associations between cognitive and affective control and adolescent mental health. Indeed, past research suggests that socioeconomic status is associated with the development of cognitive control across adolescence (e.g., Brieant et al., 2021; Li et al., 2022). However, given that our measure of socioeconomic status relied on young peoples' perceptions, replication of these associations using more objective measures of socioeconomic status will be important. Finally, the hypotheses and analysis plan were not preregistered.

Nonetheless, the present results offer important insights into the potential role of cognitive and affective control in the experience of depressive symptoms amongst adolescents, as well as mental health problems more broadly. Converging with past meta‐analytic findings showing cross‐sectional (Wagner et al., 2015) as well as prospective associations (Yang et al., 2022) between cognitive control and depressive symptoms amongst adolescents, we found that greater current depressive symptomatology was associated with reduced cognitive and affective working memory. Self‐reported diagnostic history of mental health problems was associated with poorer affective shifting ability. The findings suggest that working memory problems, in particular, may be associated with the experience of depressive symptoms in adolescents, whereas problems with affective shifting may be associated with a range of mental health problems in adolescents. Though the observed effects were small, small effects at a population level can be clinically meaningful (Carey et al., 2023). Given the ubiquitous need for efficient cognitive functioning in daily life, enhancing cognitive and affective control in adolescents may be a promising means of improving daily functioning and potentially reducing a risk factor for mental health symptomatology.

## AUTHOR CONTRIBUTIONS

**Savannah Minihan**: Data curation; formal analysis; methodology; writing – original draft; writing – review & editing. **Levi Kumle**: Data curation; methodology. **Kate Maston**: Methodology; project administration. **Debopriyo Bal**: Data curation. **Aliza Werner‐Seidler**: Funding acquisition; methodology; project administration; supervision; writing – review & editing. **Helen Christensen**: Funding acquisition; methodology; supervision; writing – review & editing. **Susanne Schweizer**: Formal analysis; methodology; supervision; writing – review & editing.

## CONFLICT OF INTEREST STATEMENT

The authors declare no conflicts of interest.

## ETHICAL CONSIDERATIONS

Ethics approvals were obtained from the University of New South Wales Human Research Ethics Committee (HC180836), the State Education Research Applications Process for the New South Wales Department of Education (SERAP2019201), and relevant Catholic Schools Dioceses across Australia. Participating schools were provided with recruitment materials (available in English, Arabic, and Chinese) to share with Year 8 families, inviting parents/guardians to provide informed consent (online, by paper, or by phone) for their child's participation. Prior to taking part in the study, all participants required informed consent from a parent or guardian.

## Supporting information

Supporting Information S1

## Data Availability

The data that support the findings of this study are available from the corresponding author upon reasonable request.
