# Supplementary material for: The relationship between cognitive and affective control and adolescent mental health
Source: JCPP Adv. 2023 Nov 3;4(1):e12204. doi: 10.1002/jcv2.12204 (PMC10933673; doi:10.1002/jcv2.12204)
Supplement: Supplementary file 1 — Supporting Information S1 [file JCV2-4-e12204-s001.docx]

**The Relationship Between Cognitive and Affective Control and Adolescent Mental Health**

**Supporting Information**

**Appendix S1. Comparison of Task Completers vs. Non-Completers**

Participants from the Future Proofing Study (Werner-Seidler et al., 2020, 2022) who completed at least one instance of one of the cognitive tasks (*n* = 1,929) did not differ to participants who did not complete either of the cognitive tasks (*n* = 4,459) on age (*M*_completers_ = 13.89, *M*_non-completers_ = 13.92, *t* = 1.84, *p* = .065), perceived socioeconomic status (*M*_completers_ = 2.45, *M*_non-completers_ = 2.42, *t* = −1.43, *p* = .152), or self-reported diagnostic history of mental health problems (*χ^2^* = 1.53, *p* = .216). However, depressive symptoms were significantly higher in completers (*M* = 7.86) compared to non-completers (*M* = 7.14; *t* = −4.18, *p* < .001). Task completion vs. non-completion was also significantly associated with gender identity (*χ^2^* = 68.90, *p* < .001; see Table S1).

**Table S1**

***Task Completion vs. Non-Completion as a Function of Gender Identity***

|  | **Female** | **Male** | **Non-Binary** | **Other** | **Prefer Not to Say** |
| --- | --- | --- | --- | --- | --- |
| **Task Completers**  Observed  Expected | 1062  942.76 | 751  897.46 | 46  35.33 | 30  20.53 | 40  32.92 |
| **Task Non-Completers**  Observed  Expected | 2060  2179.24 | 2221  2074.54 | 71  81.67 | 38  47.47 | 69  76.09 |

**Appendix S2. Task Conditions Completed Across Sessions One and Two**

For the backward digit-span task, 224 participants completed both the cognitive and affective control conditions in session 1 and 66 participants in session 2; 646 participants completed only the affective control condition in session 1 and 354 participants in session 2; 715 participants completed only the cognitive control condition in session 1 and 300 participants in session 2. For the set-shifting task, 298 participants completed both the cognitive and affective control conditions of the task in session 1 and 78 participants in session 2; 417 participants completed only the affective control condition in session 1 and 368 participants in session 2; 720 participants completed only the cognitive control condition in session 1 and 243 participants in session 2.

**Appendix S3. Statistical Analyses**

All analyses were conducted in R Studio version 4.1.2. using the psych package (Revelle, 2021), the tidyverse package (Wickham et al., 2019), the afex package (Singmann et al., 2021), the sjPlot package (Lüdecke, 2018), the emmeans package (Lenth et al., 2023), the parameters package (Lüdecke et al., 2020), and the apatables package (Stanley, 2022).

**Table S2**

***Sensitivity Analyses of the Relationship Between Cognitive and Affective Working Memory and Depressive Symptoms***

|  | **Model 1: Working Memory** | | | | **Model 2: Working Memory** | | | | **Model 3: Working Memory** | | | |
| --- | --- | --- | --- | --- | --- | --- | --- | --- | --- | --- | --- | --- |
| *Coefficient* | *b* | *SE* | *95% CI* | *p* | *b* | *SE* | *95% CI* | *p* | *b* | *SE* | *95% CI* | *p* |
| Intercept | 3.99 | 0.68 | 2.67 – 5.32 | <.001 | 3.89 | 0.07 | 3.75 – 4.03 | <.001 | 3.70 | 0.13 | 3.44 – 3.96 | <.001 |
| Condition | −0.05 | 0.02 | −0.09 – −0.00 | .039 | −0.05 | 0.02 | −0.09 – −0.00 | .037 | −0.05 | 0.02 | −0.10 – −0.00 | .040 |
| Session Number | **0.25** | **0.05** | **0.15 – 0.34** | **<.001** | **0.25** | **0.05** | **0.15 – 0.34** | **<.001** | **0.23** | **0.05** | **0.13 – 0.33** | **<.001** |
| Depressive Symptoms | **−0.01** | **0.00** | **−0.02 – −0.00** | **.004** | −0.01 | 0.00 | −0.02 – −0.00 | .013 | −0.01 | 0.00 | −0.02 – −0.00 | .019 |
| Condition x Depressive Symptoms | −0.00 | 0.00 | −0.01 – 0.00 | .269 | −0.00 | 0.00 | −0.01 – 0.00 | .247 | −0.01 | 0.00 | −0.01 – 0.00 | .069 |
| Age | −0.01 | 0.05 | −0.10 – 0.09 | .886 |  |  |  |  |  |  |  |  |
| Gender Identity (Male) |  |  |  |  | 0.02 | 0.06 | −0.09 – 0.14 | .678 |  |  |  |  |
| Gender Identity (Non-Binary) |  |  |  |  | 0.14 | 0.18 | −0.21 – 0.49 | .426 |  |  |  |  |
| Gender Identity (Other) |  |  |  |  | −0.01 | 0.22 | −0.44 – 0.41 | .962 |  |  |  |  |
| Gender Identity (Prefer Not to Say) |  |  |  |  | −0.25 | 0.18 | −0.61 – 0.11 | .179 |  |  |  |  |
| Perceived SES |  |  |  |  |  |  |  |  | 0.10 | 0.05 | 0.01 – 0.19 | .023 |
| **Random Effects** | | | | | | | | | | | | |
| σ^2^ | 1.10 | | | | 1.10 | | | | 1.10 | | | |
| τ_00_ | 0.45 _ID_ | | | | 0.45 _ID_ | | | | 0.46 _ID_ | | | |
| ICC | .29 | | | | .29 | | | | .29 | | | |
| N | 1639 _ID_ | | | | 1639 _ID_ | | | | 1438 _ID_ | | | |
| Observations | 2564 | | | | 2564 | | | | 2272 | | | |
| Marginal R^2^ / Conditional R^2^ | .014 / 0.302 | | | | .015 / 0.302 | | | | .017 / 0.306 | | | |

*Note.* Table S2 shows the results of sensitivity analyses of the relationship between cognitive and affective working memory and depressive symptoms. Working memory was operationalized as max span level achieved on a backward digit-span task (Schweizer et al., 2019). The task included a cognitive control condition, in which digits were superimposed over neutral images, and an affective control condition, in which digits were superimposed over negative images. This variable was contrast-coded for analyses: cognitive control condition = −1, affective control condition = 1. Depressive symptoms were assessed with the 9-item PHQ-A and were mean-centered for analyses; higher scores indicate greater depressive symptomatology (Johnson et al., 2002). Sensitivity analyses controlling for age, gender identity, and perceived socioeconomic status were conducted. Age was modelled numerically. Gender identity was dummy-coded, with female as the reference level. Perceived socioeconomic status was modelled numerically, where 1 = low, 2 = medium, 3 = high, NA = prefer not to say.

**Table S3**

***Sensitivity Analyses of the Relationship Between Cognitive and Affective Shifting and Self-Reported Diagnostic History of Mental Health Problems***

|  | **Model 1: Shifting Ability** | | | | **Model 2: Shifting Ability** | | | | **Model 3: Shifting Ability** | | | |
| --- | --- | --- | --- | --- | --- | --- | --- | --- | --- | --- | --- | --- |
| *Coefficient* | *b* | *SE* | *95% CI* | *p* | *b* | *SE* | *95% CI* | *p* | *b* | *SE* | *95% CI* | *p* |
| Intercept | 0.45 | 0.15 | 0.17 – 0.74 | .002 | 0.45 | 0.01 | 0.42 – 0.47 | <.001 | 0.47 | 0.03 | 0.42 – 0.53 | <.001 |
| Condition | 0.01 | 0.01 | 0.00 – 0.02 | .032 | 0.01 | 0.01 | −0.00 – 0.02 | .059 | 0.01 | 0.01 | −0.00 – 0.02 | .104 |
| Session Number | **−0.08** | **0.01** | **−0.10 – −0.07** | **<.001** | **−0.08** | **0.01** | **−0.10 – −0.07** | **<.001** | **−0.08** | **0.01** | **−0.10 – −0.07** | **<.001** |
| Self-Reported Diagnostic History | 0.01 | 0.01 | −0.01 – 0.02 | .267 | 0.01 | 0.01 | −0.00 – 0.03 | .122 | 0.01 | 0.01 | −0.01 – 0.02 | .371 |
| Condition x Self-Reported Diagnostic History | **0.01** | **0.01** | **0.00 – 0.03** | **.011** | 0.01 | 0.01 | 0.00 – 0.02 | .017 | 0.01 | 0.01 | 0.00 – 0.02 | .035 |
| Age | 0.00 | 0.01 | −0.02 – 0.02 | .919 |  |  |  |  |  |  |  |  |
| Gender Identity (Male) |  |  |  |  | **0.06** | **0.01** | **0.04 – 0.09** | **<.001** |  |  |  |  |
| Gender Identity (Non-Binary) |  |  |  |  | −0.06 | 0.04 | −0.13 – 0.01 | .096 |  |  |  |  |
| Gender Identity (Other) |  |  |  |  | 0.01 | 0.05 | −0.08 – 0.10 | .885 |  |  |  |  |
| Gender Identity (Prefer Not to Say) |  |  |  |  | 0.05 | 0.04 | −0.03 – 0.13 | .233 |  |  |  |  |
| Perceived SES |  |  |  |  |  |  |  |  | −0.00 | 0.01 | −0.02 – 0.01 | .614 |
| **Random Effects** | | | | | | | | | | | | |
| σ^2^ | 0.03 | | | | 0.03 | | | | 0.03 | | | |
| τ_00_ | 0.03 _ID_ | | | | 0.03 _ID_ | | | | 0.03 _ID_ | | | |
| ICC | .48 | | | | .48 | | | | .47 | | | |
| N | 1406 _ID_ | | | | 1406 _ID_ | | | | 1242 _ID_ | | | |
| Observations | 2316 | | | | 2316 | | | | 2058 | | | |
| Marginal R^2^ / Conditional R^2^ | .026 / 0.498 | | | | .046 / 0.500 | | | | .027 / 0.480 | | | |

*Note.* Table S3 shows the results of sensitivity analyses of the relationship between cognitive and affective shifting and self-reported diagnostic history of mental health problems. Shifting ability was operationalized as proportion of random errors on a card-sorting task, with higher scores indicating poorer shifting ability (Schweizer et al., 2019). The task included a cognitive control and affective control condition, with task condition manipulated by inclusion of a sorting rule according to shape (cognitive control condition) or emotional expression (affective control condition). This variable was contrast-coded for analyses: cognitive control condition = −1, affective control condition = 1. Self-reported diagnostic history was assessed with a multi-response item, where participants indicated whether they had ever been diagnosed by a professional with a mental health problem, selecting all the apply from the following: major depression, social anxiety disorder/social phobia, generalized anxiety disorder, obsessive compulsive disorder, panic disorder, separation anxiety disorder, alcohol use disorder, substance use disorder, attention deficit hyperactivity disorder, post-traumatic stress disorder, schizophrenia/psychosis, none of the above. This variable was contrast-coded for analyses: −1 = participant reported no mental health diagnoses; 1 = participant reported having been diagnosed with a mental health problem. Session number was modelled numerically (session one = 1, session two = 2). Sensitivity analyses controlling for age, gender identity, and perceived socioeconomic status were conducted. Age was modelled numerically. Gender identity was dummy-coded, with female as the reference level. Perceived socioeconomic status was modelled numerically, where 1 = low, 2 = medium, 3 = high, NA = prefer not to say.

**Table S4**

***The Relationship Between Cognitive and Affective Shifting and Self-Reported Diagnostic History of Mental Health Problems: Speed-Accuracy Trade-Off Analyses***

|  | **Model 1: Shifting Ability** | | | | **Model 2: Shifting Ability** | | | |
| --- | --- | --- | --- | --- | --- | --- | --- | --- |
|  | *b* | *SE* | *95% CI* | *p-value* | *b* | *SE* | *95% CI* | *p-value* |
| Intercept | 2639.07 | 72.18 | 2497.51 – 2780.62 | **<.001** | 2663.68 | 75.76 | 2515.11 – 2812.25 | **<.001** |
| Condition | **328.47** | **24.03** | **281.35 – 375.60** | **<.001** | **337.95** | **32.32** | **274.57 – 401.33** | **<.001** |
| Session Number | **−229.89** | **52.34** | **−332.54 – −127.24** | **<.001** | **−229.22** | **52.35** | **−331.87 – −126.57** | **<.001** |
| Self-Reported Diagnostic History |  |  |  |  | 37.69 | 34.88 | −30.71 – 106.09 | .280 |
| Condition x Self-Reported Diagnostic History |  |  |  |  | 13.62 | 32.15 | −49.43 – 76.66 | .672 |
| σ^2^ | 1174513.05 | | | | 1174420.97 | | | |
| τ_00_ | 173527.45 _ID_ | | | | 172807.05 _ID_ | | | |
| ICC | .13 | | | | .13 | | | |
| N | 1394 _ID_ | | | | 1394 _ID_ | | | |
| Observations | 2273 | | | | 2273 | | | |
| Marginal R^2^ / Conditional R^2^ | .074 / 0.193 | | | | .074 / 0.193 | | | |

*Note.* Table S4 shows the results of two linear mixed effects models investigating the potential of a speed-accuracy trade-off in the relationship between cognitive and affective shifting and self-reported diagnostic history of mental health problems. Shifting ability was operationalized as reaction time on random error trials on a card-sorting task (Schweizer et al., 2019). The task included a cognitive control and affective control condition, with task condition manipulated by inclusion of a sorting rule according to shape (cognitive control condition) or emotional expression (affective control condition). This variable was contrast-coded for analyses: cognitive control condition = −1, affective control condition = 1. Self-reported diagnostic history was assessed with a multi-response item, where participants indicated whether they had ever been diagnosed by a professional with a mental health problem, selecting all the apply from the following: major depression, social anxiety disorder/social phobia, generalized anxiety disorder, obsessive compulsive disorder, panic disorder, separation anxiety disorder, alcohol use disorder, substance use disorder, attention deficit hyperactivity disorder, post-traumatic stress disorder, schizophrenia/psychosis, none of the above. This variable was contrast-coded for analyses: −1 = participant reported no mental health diagnoses; 1 = participant reported having been diagnosed with a mental health problem. Session number was modelled numerically (session one = 1, session two = 2).

**References**

Johnson, J. G., Harris, E. S., Spitzer, R. L., & Williams, J. B. W. (2002). The patient health questionnaire for adolescents: Validation of an instrument for the assessment of mental disorders among adolescent primary care patients. *Journal of Adolescent Health*, *30*(3), 196–204. https://doi.org/10.1016/S1054-139X(01)00333-0

Lenth, R. V., Buerkner, P., Giné-Vázquez, I., Herve, M., Jung, M., Love, J., Miguez, F., Riebl, H., & Singmann, H. (2023). *emmeans: Estimated Marginal Means, aka Least-Squares Means* (1.8.4-1) [Computer software]. https://CRAN.R-project.org/package=emmeans

Lüdecke, D. (2018). sjPlot: Data visualization for statistics in social science. *R Package Version*, *2*(1).

Lüdecke, D., Ben-Shachar, M., Patil, I., & Makowski, D. (2020). Extracting, Computing and Exploring the Parameters of Statistical Models using R. *Journal of Open Source Software*, *5*(53), 2445. https://doi.org/10.21105/joss.02445

Revelle, W. (2021). *psych: Procedures for Psychological, Psychometric, and Personality Research* (2.1.9) [Computer software]. https://CRAN.R-project.org/package=psych

Schweizer, S., Leung, J. T., Kievit, R., Speekenbrink, M., Trender, W., Hampshire, A., & Blakemore, S.-J. (2019). Protocol for an app-based affective control training for adolescents: Proof-of-principle double-blind randomized controlled trial. *Wellcome Open Research*, *4*. https://doi.org/10.12688/wellcomeopenres.15229.2

Singmann, H., Bolker, B., Westfall, J., Aust, F., Ben-Shachar, M. S., Højsgaard, S., Fox, J., Lawrence, M. A., Mertens, U., Love, J., Lenth, R., & Christensen, R. H. B. (2021). *afex: Analysis of Factorial Experiments* (1.0-1) [Computer software]. https://CRAN.R-project.org/package=afex

Stanley, D. (2022). *Package ‘apaTables.’* https://cran.r-project.org/web/packages/apaTables/apaTables.pdf

Werner-Seidler, A., Huckvale, K., Larsen, M. E., Calear, A. L., Maston, K., Johnston, L., Torok, M., O’Dea, B., Batterham, P. J., Schweizer, S., Skinner, S. R., Steinbeck, K., Ratcliffe, J., Oei, J.-L., Patton, G., Wong, I., Beames, J., Wong, Q. J. J., Lingam, R., … Christensen, H. (2020). A trial protocol for the effectiveness of digital interventions for preventing depression in adolescents: The Future Proofing Study. *Trials*, *21*(1), 2. https://doi.org/10.1186/s13063-019-3901-7

Werner-Seidler, A., Maston, K., Calear, A. L., Batterham, P., Larsen, M., Torok, M., O’Dea, B., Huckvale, K., Beames, J. R., & Brown, L. (2022). *The Future Proofing Study: Design, Methods and Baseline Characteristics of a Prospective Cohort Study of the Mental Health of Australian Adolescents*.

Wickham, H., Averick, M., Bryan, J., Chang, W., McGowan, L. D., François, R., Grolemund, G., Hayes, A., Henry, L., Hester, J., Kuhn, M., Pedersen, T. L., Miller, E., Bache, S. M., Müller, K., Ooms, J., Robinson, D., Seidel, D. P., Spinu, V., …

Yutani, H. (2019). Welcome to the Tidyverse. *Journal of Open Source Software*, *4*(43), 1686. https://doi.org/10.21105/joss.01686
